# Limitations in a frataxin knockdown cell model for Friedreich ataxia in a high-throughput drug screen

**DOI:** 10.1186/1471-2377-9-46

**Published:** 2009-08-24

**Authors:** Nadège Calmels, Hervé Seznec, Pascal Villa, Laurence Reutenauer, Marcel Hibert, Jacques Haiech, Pierre Rustin, Michel Koenig, Hélène Puccio

**Affiliations:** 1 IGBMC (Institut de Génétique et de Biologie Moléculaire et Cellulaire), CNRS/INSERM/Université Louis Pasteur, 67404 Illkirch cedex, France; 2 IFR 85/PCBIS (Plateforme de Chimie Biologique Intégrative de Strasbourg), ESBS Pôle API, 67401 Illkirch, France; 3 UMR7175/CNRS/Université Louis Pasteur, 67404 Illkirch cedex, France; 4 INSERM U676, Hôpital Robert Debré, 75019 Paris, France; 5 Interface Physique Biologie, Centre d'Etudes Nucléaires de Bordeaux-Gradignan, CNRS/IN2P3-UMR5797, BP120 - 33175 Gradignan, France

## Abstract

**Background:**

Pharmacological high-throughput screening (HTS) represents a powerful strategy for drug discovery in genetic diseases, particularly when the full spectrum of pathological dysfunctions remains unclear, such as in Friedreich ataxia (FRDA). FRDA, the most common recessive ataxia, results from a generalized deficiency of mitochondrial and cytosolic iron-sulfur cluster (ISC) proteins activity, due to a partial loss of frataxin function, a mitochondrial protein proposed to function as an iron-chaperone for ISC biosynthesis. In the absence of measurable catalytic function for frataxin, a cell-based assay is required for HTS assay.

**Methods:**

Using a targeted ribozyme strategy in murine fibroblasts, we have developed a cellular model with strongly reduced levels of frataxin. We have used this model to screen the Prestwick Chemical Library, a collection of one thousand off-patent drugs, for potential molecules for FRDA.

**Results:**

The frataxin deficient cell lines exhibit a proliferation defect, associated with an ISC enzyme deficit. Using the growth defect as end-point criteria, we screened the Prestwick Chemical Library. However no molecule presented a significant and reproducible effect on the proliferation rate of frataxin deficient cells. Moreover over numerous passages, the antisense ribozyme fibroblast cell lines revealed an increase in frataxin residual level associated with the normalization of ISC enzyme activities. However, the ribozyme cell lines and FRDA patient cells presented an increase in Mthfd2 transcript, a mitochondrial enzyme that was previously shown to be upregulated at very early stages of the pathogenesis in the cardiac mouse model.

**Conclusion:**

Although no active hit has been identified, the present study demonstrates the feasibility of using a cell-based approach to HTS for FRDA. Furthermore, it highlights the difficulty in the development of a stable frataxin-deficient cell model, an essential condition for productive HTS in the future.

## Background

In the past 20 years, many genes involved in different rare genetic disorders have been identified. However, many causative genes encode proteins of unknown or partially known function, and with no predictable catalytic sites enabling *in vitro *drug-target modeling. This makes drug design and identification a difficult task for these orphan diseases. However, new strategies based on automated techniques for high-throughput screening (HTS) have been developed, enabling cell-based assays to identify drug-candidates.

Friedreich's ataxia (FRDA), the most common autosomal recessive ataxia associating spinocerebellar ataxia and cardiomyopathy [1,2], is most often due to a (GAA)_n _repeat expansion within the first intron of the gene encoding the mitochondrial protein frataxin [3,4]. This frequent mutation leads to a severely reduced level of frataxin as a consequence of transcriptional silencing either through heterochromatin formation or through the formation of a triplex helix [5-7]. Although much progress has been made in understanding the physiopathology of FRDA, the exact role of the frataxin protein is still unclear. Early studies showed iron deposits in cardiac tissue of FRDA patients [8] and in the yeast strain deleted for frataxin (Δ*YFH1*) thereby linking impaired iron homeostasis to the disease [9]. This led to the hypothesis that elevated levels of mitochondrial iron, as a consequence of frataxin deficiency, could generate cell-damaging superoxide and hydroxyl radicals through Fenton reaction. In support of this, several studies have suggested an increased levels of oxidative stress in patients [10-13], as well as an increased sensitivity to oxidative stress in FRDA patients cells or Δ*YFH1*-yeast model [14-18]. However, more recent studies have found no evidence of increased oxidative damage in FRDA patients [19-21]. Moreover, experimental data from the conditional FRDA mouse models demonstrated that an increased superoxide production could not explain by itself the FRDA pathology [22] and that the mitochondrial iron accumulation is a late event in the disease [23]. In different mouse and cellular models, the primary biochemical event is the impaired function of iron-sulfur cluster (ISC) proteins such as the aconitases and respiratory chain complexes I-III [23-26]. The role of frataxin as a mitochondrial iron-chaperone for ISC biogenesis is now widely accepted. In addition to severe alteration of mitochondrial and extramitochondrial ISC proteins in frataxin-deficient yeast, mice or human cells [23,27-30], reconstitutional and *in vivo *studies demonstrate that the yeast frataxin homolog Yfh1 is required, although not essential, for ISC biosynthesis [27,29]. Furthermore, frataxin has been demonstrated to interact with the ISC biosynthesis scaffold complex IscU/Nfs1/ISD11 [31-35].

Several therapeutic strategies for FRDA have been developed based on the potential implication of these different pathways in the pathogenesis. Some pharmacological compounds such as antioxidants ([36] for review) or iron chelators [37-39] have shown promise in improving some of the symptoms of the disease. Recently, new therapeutic strategies have been developed to address the frataxin deficiency itself: pharmacological compounds increasing frataxin protein levels (such as recombinant human erythropoietin) or reversing frataxin gene silencing (such as histone deacetylase inhibitors) have been successfully tested in clinical or preclinical trials [40,41]. However there is currently no effective pharmacological treatment available that would slow down the neurological progression of the disease in affected FRDA patients.

The development of a cellular model which reproduces accurately the major aspects of the pathogenesis is the preliminary condition before being able to make a pharmacological screen. To date, there is no appropriate mammalian cell model for FRDA. Indeed, no easily available patient's tissue or cell lines spontaneously express the generalized ISC enzyme deficiency, and exogenous stress conditions have to be used in order to reveal a differential phenotype with control fibroblasts [14,17,18,42]. Moreover, the reproducibility and relevance of the results obtained with such systems has been contested [10]. More recently, RNA interference strategies have been developed to reproduce frataxin deficiency in mammalian cell lines [16,25,43-47]. Both transient and stable frataxin silencing to undetectable levels in HeLa cells lead to significant reduction of cell growth and activities of ISC proteins [45,47]. Although it seems evident that transient frataxin silencing is not suitable for HTS experiments, it is unclear whether the stable frataxin silencing clone in HeLa cells is an appropriate model for HTS experiments as the clone is reported to have roundish and grainy cells that easily detached from the plate [47]. The RNAi models have been studied to unravel consequences of frataxin deficiency, but no HTS on a FRDA cell-model has yet been published.

In this study, we have developed and characterized a cellular model with partial frataxin deficiency using targeted ribozyme strategy. This model displayed a specific ISC deficit, faithfully and spontaneously reproducing a key feature of the human disease. The growth delay of the frataxin-deficient clone was used as a quantifiable parameter to screen the Prestwick Chemical Library for potential drug-candidates. However, the absence of confirmed active hit and the instability of the cellular model illustrate the difficulty to identify drug-candidates in a small compound library and in accurately replicating the FRDA pathogenesis in a long-term controlled cellular model.

## Methods

### Ribozyme construction

The pZeoFxnR2, pZeoFxnR2^m ^and pZeoFxnR5 vectors were constructed using the pZeoU1*EcoSpe *vector [48,49]. Complementary oligonucleotides that encode the antisense sequence, including the 24 highly conserved nucleotides of hammerhead ribozymes (underlined in the sequences below) flanked by 24 and 15 nucleotides of murine frataxin sequence in exon 2 for R2, and by 19 and 20 nucleotides of murine frataxin sequence in 3'-UTR at the end of exon 5 for R5, were synthesized and annealed at 42°C. The sequences of the oligonucleotides were as follows: R2: 5'-AAT TCC TCA AAT GCA CCA CGC AGA **CTG ATG AGT CCG TGA GGA CGA AAC **GCT CTG CTT TTT GAT-3' and 5'-CTA GAT CAA AAA GCA GAG C**GT TTC GTC CTC ACG GAC TCA TCA G**TC TGC GTG GTG CAT TTG AGG-3' (sense and antisense, respectively); R5: 5'-AAT TAG GAG CAG GTA TGG GAA GGC AGA CTG ATG AGT CCG TGA GGA CGA AAC ATT CAG CTA CAG G-3' and 5'-CTA GCC TGT AGC TGA ATG TTT CGT CCT CAC GGA CTC ATC AGT CTG CCT TCC CAT ACC TGC TCC T-3' (sense and antisense, respectively). For the mutated ribozyme R2^m^, the sequences of the oligonucleotides were as follows: R2^m^: 5'-AAT TCC TCA AAT GCA **AA**A CGC AGA **CTG ATG AGT CCG TGA GGA CGA AAC **GCT CT**T A**TT TTT GAT-3' and 5'-CTA GAT CAA AAA **TA**A GAG C**GT TTC GTC CTC ACG GAC TCA TCA G**TC TGC GT**T T**TG CAT TTG AGG-3' (sense and antisense, respectively), which include four mismatches in the two frataxin complementary sequences of exon 2 (bold in the primer sequences). The resulting duplexes were ligated into the *Eco*RI and *Spe*I sites of pZeoU1*EcoSpe *to create pZeoFxnR2 and pZeoFxnR2^m^. All ligation junctions were sequenced to verify the identity and orientation of the insert.

### Stable transfection of Frda^L2/L- ^cells

Murine fibroblast cell lines derived from mice carrying the wild type (*Frda*^+/+^), conditional (*Frda*^+/L3^) or compound heterozygous for the deleted and conditional (*Frda*^L3/L-^) frataxin alleles [23] were established using the primary-explant technique [50], and then immortalized by transfection with a Large Antigen T construct [51] using the Fugene 6 Transfection Reagent kit (Roche, Indianapolis, Indiana), according to the manufacturer's protocol. As the mice also expressed an inducible recombinase (*Cre*-ER^T^) [52], the deletion of the neomycin resistance cassette was obtained by tamoxifen treatment. The resulting cell line will be noted *Frda*^L2+/L- ^in the text.

*Frda*^L2+/L- ^immortalized cells were transfected with linearized pZeoFxnR2 or pZeoFxnR2^m^. Stably transfected fibroblasts were grown in DMEM media (Sigma, Saint Louis, Missouri) with 10% fetal calf serum and 50 μg/ml gentamycin, supplemented with 250 μg/ml Zeocin (InvivoGen, San Diego, California). Zeocin selection was maintained one month. Monoclonal cell lines were obtained by dilution cloning. Aliquots of the cell line were frozen for cryopreservation in liquid nitrogen, and a new aliquot was used for each screening experiment.

### Genotyping and immunoblot analysis

Genotyping and DNA analysis were performed as previously described [23]. Immunoblot analysis using the antifrataxin antibody (R1250 purified sera, 1/1,000) were performed as previously described [22,23]. Briefly, for protein extract, fibroblast cell lines were lysed in a buffer containing 50 mM Tris-HCl pH7.8, 10% (v/v) glycerol, 1 mM EDTA, 5 mM KCl with protease inhibitor (complete, EDTA-free protease inhibitor cocktail (Roche, Basel, Switzerland)). Cell lysates were sonicated and conserved at -20°C.

### Quantitative RT-PCR (Q-RT-PCR)

Expression levels of murine genes (*Fxn, Mthfd2, Hprt*) were determined by Q-RT-PCR as previously described [22,23]. The following primers were used for amplification of human methylenetetrahydrofolate dehydrogenase 2 (*MTHFD2*) and 18S ribosome in the same conditions: *MTHFD2*, forward 5'-TGA AGA GCG AGA AGT GCT GA-3', reverse 5'-GAA TGC TCC CTG GTG AGG TA-3'. *18S*, forward, 5'-CGC CGC TAG AGG TGA AAT TC-3', reverse 5'-CTT TCG CTC TGG TCC GTC TT-3'.

### Measurement of cell proliferation rates

Fibroblasts were plated in 6-well plates at day 1. After cell detachment using trypsin, cell densities were determined daily by visual counting with a hemocytometer. Four counts were performed per well.

### Biochemical analyses

Cells were harvested in PBS and dry pellet was immediately frozen in liquid nitrogen. The activities of the respiratory chain enzyme complexes succinate cytochrome c reductase (SCCR), cytochrome c oxydase (COX), the mitochondrial and cytosolic aconitases (Aco) and the isocitrate deshydrogenase (IDH) as internal standard were measured spectrophotometrically as previously described [23,53].

### Miniaturization and screening

The Prestwick Chemical Library (Illkirch, France) which consists of 1120 drugs and bioactive natural compounds approved by the Food and Drug Administration (FDA) was screened on both R2C1 and R2^m ^cell lines in 96-well format. Chemical compounds (~5 mM) dissolved in dimethyl sulfoxide (DMSO) were stored at -20°C into 96 well plates. Just before treatment, compounds were diluted with cell culture medium (DMEM media with 10% fetal calf serum and 50 μg/ml gentamycin) into 96 well plates.

Fresh R2C1 and R2^m ^cell aliquots were thawed for each screening experiment. At the time of passage, R2C1 and R2^m ^cell suspensions were prepared in order to be seeded into 96-well plates (Greiner Bio-one, France, ref 655090) using a Biomek 2000 Laboratory Automation Workstation (Beckman Coulter inc) (100 μl/well, 10,000 cells/ml).

After sedimentation for 30 minutes, test compounds were added to the wells (100 μl per well, one compound per well) to result in a final concentration of 25 μM. Vehicle control wells contained 0.5% DMSO alone (columns 1 and 12 of each plate). At this DMSO concentration we did not detect any side effect on cells.

Cell proliferation was assessed 72 hours after cell treatment using the commercial CellTiter-Glo Luminescent Cell viability assay (Promega, Madison, WI) generating a luminescent signal directly proportional to the amount of ATP present in metabolically active cells. After incubation, 100 μl of medium was removed and replaced by 100 μl of Promega CellTiter-Glo reagent to each well using the Biomek 2000 workstation. Plates were shaken for 5 min on a plate shaker before reading luminescence on a Victor^3 ^plate reader (PerkinElmer, Norwalk, CT). Luminescence in each treated well was compared to the mean signal obtain in non-treated wells (columns 1 and 12). Positive hits were designated for any compounds with luminescent signal over three standard deviations when compared to untreated control wells. Luminescent ratio between the R2^m ^and R2C1 cell lines was also calculated to check the reproducibility of proliferation delay in the frataxin deficient clone during the experiments (when calculated for non treated cells) and to check the specificity of drug action (when calculated for treated cells).

## Results

### Partial loss of frataxin leads to a proliferation defect in frataxin ribozyme cell lines

**Figure 1:**
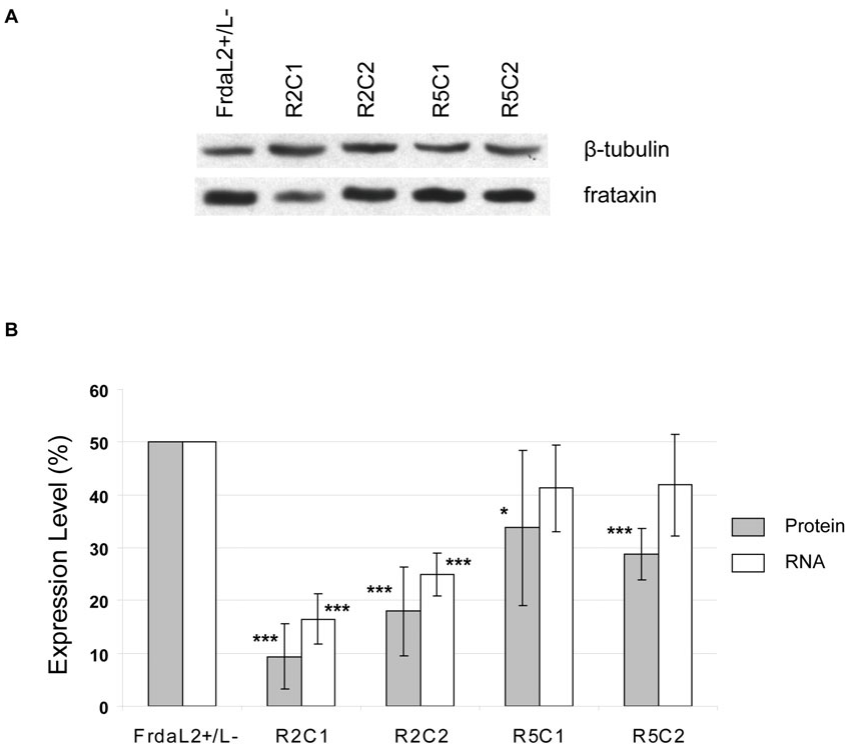
**Depletion of frataxin in ribozyme clones**. (A) Representative western blot analyses of protein extracts from different ribozymes clones revealed with polyclonal frataxin antibody is shown (five independent experiments were performed for quantitative analysis in B). Normalization was done using β-tubulin as a loading control. (B) Frataxin mRNA (white) and protein (grey) levels in antisense ribozyme fibroblast clones R2C1, R2C2, R5C1 and R5C2, compared to the Frda^L2+/L- ^non-transfected cell line. Three quantitative-RT-PCR analyses were performed on whole cellular RNA extracts, comparing frataxin expression to the reference *Hprt *gene. *p ≤ 0.05; ***p ≤ 0.005.

To generate viable FRDA cellular models with reduced frataxin level, we used a ribozyme antisense strategy in cells derived from mice carrying the conditional allele [23]. Two ribozymes, one targeted against exon 2 of the murine frataxin mRNA and one against the 3'-UTR (exon 5), were constructed in the U1snRNA based ribozyme vector pZeoSV [49]. Immortalized compound heterozygous Frda^L2+/L- ^cells were transfected with either a frataxin-specific ribozyme (pZeoFxnR2 or pZeoFxnR5) or a control construct bearing mutant frataxin sequences (pZeoFxnR2^m^). The transfected cell lines were subsequently brought through the identical selection process to generate polyclonal and monoclonal colonies of stable transfectants. Two clones were obtained with pZeoFxnR2 (R2C1 and R2C2) and four with pZeoFxnR5 (R5C1 to R5C4). The amount of frataxin was assayed by Q-RT-PCR and western blot. A better frataxin knockdown was obtained with the pZeoFxnR2 clones compared to the R5 clones (Fig. 1). Clones transfected with mutant-pZeoFxnR2^m ^showed no significant difference compared to non-transfected Frda^L2+/L- ^cells (data not shown). For subsequent experiments, we selected the two R2 clones (R2C1 and R2C2) which presented significant decrease in frataxin mRNA and protein expression levels (frataxin residual protein level: 9.4% ± 6.1 (p < 0.001) and 18.0% ± 8.4 (p < 0.001), respectively; frataxin mRNA level: 16.5% ± 4.7 (p < 0.001) and 24.9% ± 4.0 (p < 0.001), respectively) (Fig. 1B).

**Figure 2:**
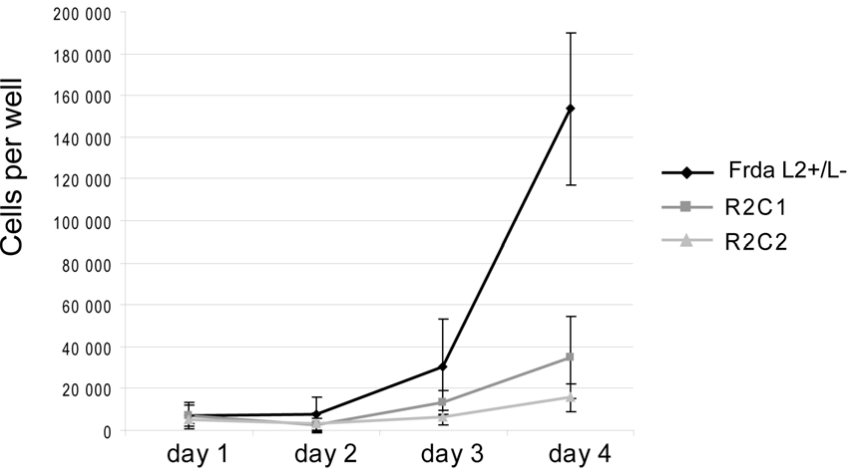
**Frataxin depletion impaired cell growth**. Growth curves on the frataxin-deficient clones R2C1 and R2C2 compared to the Frda^L2+/L- ^non-transfected cell line.

The frataxin deficient clones R2C1 and R2C2 consisted of a homogeneous cell population with no gross morphological phenotype or cell death. Electron microscopy analyses confirmed the absence of morphological phenotype, with normal mitochondria having clear thin cristae and no iron deposits (data not shown). However, growth analysis uncovered a clear proliferation defect in both frataxin deficient clones (Fig. 2). Indeed, the two clones (R2C1 and R2C2) grew slower than Frda^L2+/L- ^non-transfected cells, leading to 90% and 77% reduction of cell number, respectively, after 4 days in culture. In addition, both R2C1 and R2C2 grew slower than R2^m ^clones (see below).

### Ribozyme cell models have a significant decreased in ISC protein activities

**Figure 3:**
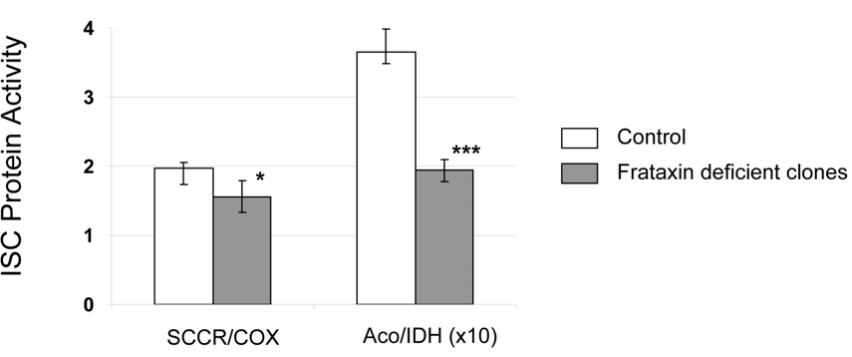
**Frataxin depletion impaired ISC-enzymes activities**. ISC-proteins activities in frataxin-deficient R2 clones (R2C1 and R2C2) and non-transfected control cell line. As the data was equivalent, data of both frataxin deficient clones were pooled for representation. SCCR (succinate cytochrome c reductase, respiratory chain complex II) and aconitases (Aco) are ISC proteins while COX (cytochrome c oxidase, respiratory chain complex IV) and IDH (isocitrate dehydrogenase) do not contain an ISC.

Frataxin deficiency in FRDA patients and in model organisms (mice, drosophila, and yeast) leads to a specific deficit in the ISC protein activities [23,30,54]. The enzymatic activities of mitochondrial respiratory chain succinate cytochrome c reductase (SCCR) and cytochrome c oxydase (COX), the tricarboxylic acid cycle enzymes aconitases (Aco), and isocitrate dehydrogenase (IDH) were measured spectrophotometrically in the frataxin-deficient clones compared to Frda^L2+/L- ^non-transfected cells. Interestingly, the two ISC enzymes (SCCR and Aco) showed a decreased activity in frataxin deficient clones (Fig. 3) with a SCCR/COX ratio of 1.56 ± 0.23 and an Aco/IDH ratio of 1.94 ± 0.16 in the frataxin deficient clones while the SCCR/COX and the Aco/IDH ratios were 1.97 ± 0.09 and 3.66 ± 0.34 in the control cells, respectively (Fig. 3). Moreover the data parallel previous results from both FRDA patients and mouse models suggesting that the aconitases are more sensitive to frataxin deficiency than SSCR. Indeed, we found a 45% decrease in activity of aconitases with only a 19% decrease in SSCR activity in the ribozyme clones.

### Medium-scale pharmacological screen of the ribozyme cell model

The proliferation delay of frataxin-deficient clones is an assay readout that is amenable to downscaling to 96-well plate format for easy automation. Set-up experiments were performed in order to select the best mutant cell line (R2C1 or R2C2), the seeding concentration (100 to 1,000 cells per well) and the time of culture before reading (3 or 4 days). Four different methods were tested to measure cell proliferation, based on nucleic acid detection (CyQUANT cell proliferation assay, Molecular Probe), ATP production (CellTiter-Glo Luminescent Cell Viability Assay, Promega) or reduction of tetrazolium salt (MTT test and CellTiter 96 AQueous Cell Proliferation Assay, Promega). The CellTiter-Glo assay showed the best results with a growth ratio (R2^m ^control cells/R2C1 frataxin deficient cells) of 1.87 (corresponding to a 47% decrease growth rate for the frataxin-deficient clone) and the smallest standard deviation after 3 days. No major decantation effect or edge effect was detected. Due to contact inhibition of the cells occurring when confluence is reached into 96-well plates, the optimal time for output readings was 72 hrs after seeding.

**Figure 4:**
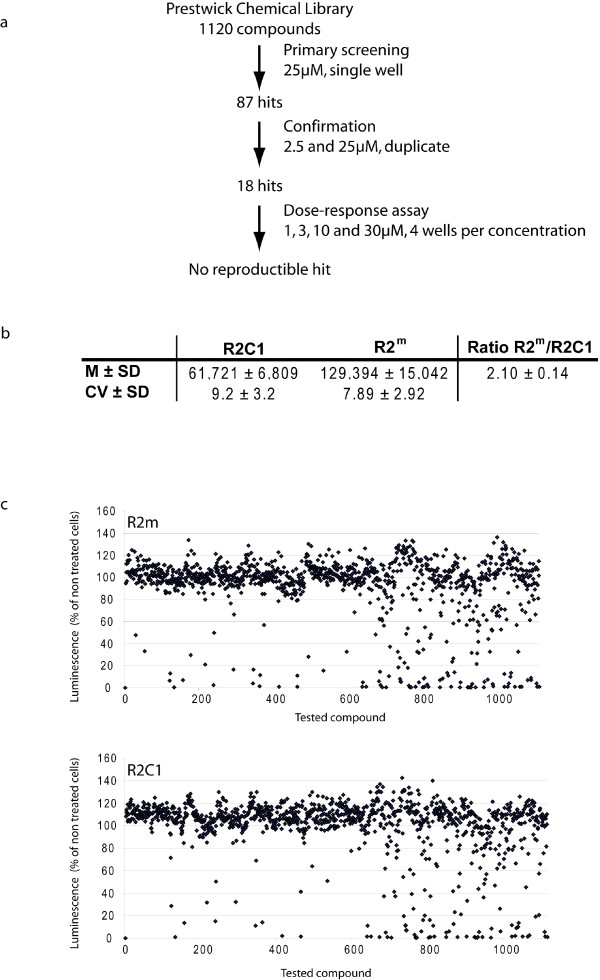
**Screening of the Prestwick Chemical Library**. (A) Schematic representation of the three-step screening with hits yields. (B) Luminescence intensity of frataxin-deficient (R2C1) and control (R2^m^) untreated cell lines during the primary screening (14 plates for each cell line). Mean (M), standard deviation (SD), coefficient of variation (CV). (C) Effect of the 1,120 compounds of the library on the luminescence intensity in the control cell line R2^m ^(upper panel) and in the frataxin-deficient clone R2C1 (lower panel) during the primary screening. Results are expressed as percentage of luminescence intensity compared to the same non treated cell line.

Using the established screening conditions, the Prestwick Chemical Library, a collection of off-patent drugs and alkaloids, was screened to find candidate pharmacological compounds that could rescue the proliferation defect of the frataxin deficient cell line. The 1,120 molecules of the library were tested at a single concentration of 25 μM in 96-well format (Fig. 4A) using the CellTiter-Glo luminescent cell viability assay. We performed the screen on both frataxin deficient (R2C1) and control (R2^m^) cell lines. The Z'-factor classically used in evaluation and validation of HTS assays [55] cannot be evaluated in this screen as positive controls on cell growth were not available. The statistical analysis of luminescence intensity for frataxin-deficient (R2C1) and control (R2^m^) untreated cell lines during the primary screening showed a good growth ratio with small standard deviation (Fig. 4B). The coefficients of variation (CV) are within the acceptable range for a cell-based assay; i.e. below 10%. The control cell line proliferation rate was 2.10 ± 0.14 times higher when compared to the frataxin-deficient clone. Positive hits were designated for any compound with cellular growth increment over three standard deviations (increase in luminescence intensity as compared to untreated control wells of the same cell line). Eighty-seven primary hits were identified in the primary screening: fifty molecules increased proliferation rate in frataxin deficient cell line only, forty-six in the control cell line only and nine molecules had proliferative effect on both cell lines (Fig. 4C). All eighty-seven primary hits were tested for confirmation at two concentrations (2.5 and 25 μM) in duplicate, using the same hit selection criteria. Eighteen hits were retained with proliferative though minor effect, and were followed-up in a dose-response analysis. Each compound was tested at five concentrations (1, 3, 10, 30 and 100 μM) with four wells per concentration. However at this stage, no molecule presented a significant and reproducible strong effect on the proliferation rate of the frataxin deficient cell line (Fig. 4A).

### Instability of the frataxin deficiency in the ribozyme cell lines

**Figure 5:**
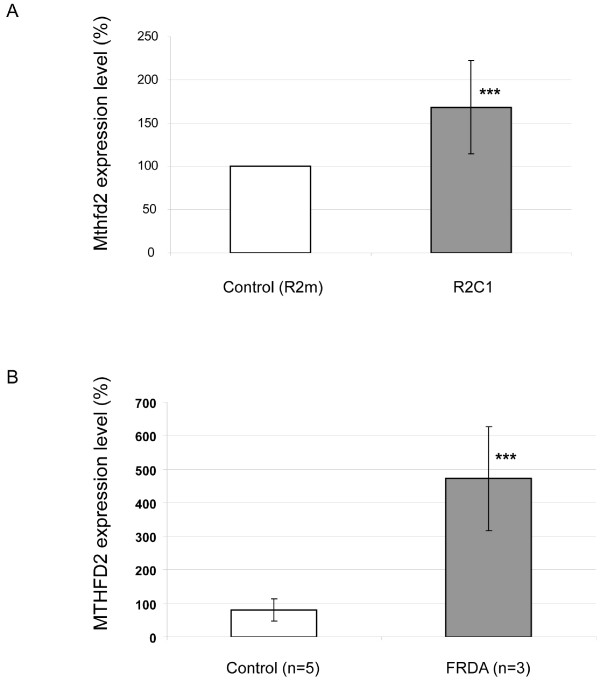
**Mthfd2 expression level in ribozyme clones and in FRDA patient's fibroblasts**. (A) Quantitative-RT-PCR analyses were performed on three different cellular RNA extracts of R2^m ^and R2C1 clones, comparing the expression of murine Mthfd2 transcript to the reference Hprt transcript. The R2C1 cells used for these experiments present the "adjusted" frataxin residual level of 29 ± 7%. (B). Quantitative-RT-PCR analyses were performed on two different cellular RNA extracts of FRDA (n = 3) and control (n = 5) fibroblasts, comparing expression of the human MTHFD2 transcript to 18S ribosome transcript. ***p ≤ 0.005.

It is important to note that the R2C1 ribozyme cell line underwent numerous passages over a period of two years during the entire model set up and screening development. We therefore were interested in testing the stability of frataxin deficiency on different aliquots throughout the screening period. Despite the presence of the ribozyme (both at the genomic and RNA level, data not shown), Q-RT-PCR demonstrated that the frataxin mRNA level had increased by 1.8 fold (with a basal frataxin expression from 16 ± 5% to 29 ± 7% of wild type level). This "adjusted level" of frataxin deficiency was still associated with a deficit in the proliferation rate (by manual counting: 1,97 ratio at day 3 and 65% reduction of cell number at day 4), but the ISC protein activities (SCCR and aconitases) deficit was no longer present. The R2C1 cell line therefore became similar to FRDA patient's fibroblasts where no ISC enzyme deficiency can be found. However, Q-RT-PCR demonstrated that the "adjusted level" R2C1 cell line, in addition to the proliferation deficit, presented an increase of the Mthfd2 transcript (Fig. 5A), a transcriptional change associated with frataxin deficiency in mouse models [22] and FRDA fibroblasts (Fig. 5B).

## Discussion

Cellular models are of great value for drug screening strategies and often represent an essential tool for both investigating molecular mechanisms of genetic diseases and identifying pharmacologically active compounds. We have used an antisense ribozyme strategy to establish a cell line with reduced frataxin level. Two clones presented a significant decrease in frataxin with a clear decrease in cell proliferation. Moreover, these frataxin deficient cells initially showed a deficit in the activity of two ISC enzymes (45% decrease in aconitase activity and 19% decrease in SCCR activity), a specific and characteristic feature of the human disease.

Our results on the ribozyme cell lines are in agreement with data observed in both transient and stable frataxin silencing models in HeLa cells obtained by RNAi strategies [45,47]. Upon frataxin depletion below ~20%, the three models display reduction of growth and decreased activity of the ISC proteins, specifically aconitases and succinate dehydrogenase. However, one notable difference is the altered morphology observed in the stable RNAi HeLa cell line, with roundish and grainy cells that easily detached from the plate [47]. This abnormal morphology is most likely due to the strongly reduced level of frataxin (almost undetectable) in the stable RNAi HeLa model compared to the normal phenotype of cells presenting approximately 10 to 20% of residual frataxin ([45] and this manuscript). Indeed, no clone with a frataxin level below 9% was found in the present study. In agreement with these results, we have recently shown that complete absence of frataxin in murine fibroblasts inhibits cell division and leads to cell death (unpublished results). These observations suggest that a threshold level of frataxin is necessary for cell proliferation and survival, consistent with the early embryonic lethality of the classical knock-out mouse model [56].

While some candidate-based pharmacological compounds such as antioxidants ([36] for review) or iron chelators [37] have shown some promising results in clinical trial in providing protection on certain aspects of the disease, there is still no effective therapy for FRDA. Moreover clinical drug trials are difficult to organize for a disease like FRDA, due to the slowly progressive and chronic nature of the disease, the very large individual variations (due in part to the unstable expansion mutation), the low frequency of the disease, and the ethical issues raised by double-blind testing. Cellular models of the human disease short-cut all these problems, and should allow to obtain faster and more reliable results on efficacy of new compounds in pre-clinical trials. We have therefore used our frataxin-deficient cell line to perform a pharmacological robotized screening. Indeed, HTS has revealed to be a successful strategy for drug discovery, especially when rational design based on the knowledge of the molecular cause of the pathological dysfunction is unclear. To circumvent the limitations of screening large libraries, we have chosen to study a limited number of already available drug molecules, in accordance to the "selective optimization of side activities" (SOSA) approach [57]. Indeed, the Prestwick Chemical Library consists in 1,120 compounds that are structurally and therapeutically very diverse with known safety and bioavailability in humans. Despite the identification of 87 primary hits, 18 of which were confirmed at the secondary step, no molecule of the library has revealed a significant and reproducible positive effect on cell growth of the frataxin-deficient clone on a dose-response curve. This failure may be due to the rather aspecific assay readout chosen to follow the efficiency of the molecules. Indeed, cell proliferation is under the control of multiple regulatory pathways and could lead to high rate of false positive and negative results. The activities of ISC proteins would probably be a more suitable end-point for FRDA screening but these enzymatic measurements are not available for automated quantification. A second explanation to this unproductive screen could be the rather low number of tested molecules. Knowing that such screening strategies retrieves between 0.1 and 1% of hits, it is statistically not surprising not to find a hit in a 1,120 compounds library. The screen of a larger compound library could allow to find active compounds. In spite of the absence of positive hit, this first cell-based automated screen on a FRDA cell model demonstrates the feasibility of the strategy.

The primary screen also identified one hundred and fifty one molecules decreasing ATP production in frataxin deficient and/or control cell lines. Two molecules specifically reduced the proliferation rate of the frataxin deficient clone. Although at first sight, the mecanism of action of both molecules (one expectorant and one vasodilatator) is hard to link to the current knowledge on frataxin, further investigation may uncover new pathways linked to frataxin.

Long term maintenance of the R2C1 frataxin-deficient clone has revealed a cellular adaptation to frataxin deficiency. Despite the persistence of the ribozyme construction, frataxin mRNA level has increased from 16 to 29% by an unknown compensatory mechanism, either by an increase in transcription or stability of the endogenous Fxn transcript, or by a reduction of the ribozyme efficiency. This slight increase in frataxin level was sufficient to restore normal ISC enzyme activities whereas proliferative deficit of the frataxin-deficient clone was still present. Furthermore, the Mthfd2 transcript, a mitochondrial enzyme involved in reduction of folate cofactors, was increased in the R2C1 clone, as previously observed at very early stages of pathogenesis in the cardiac mouse models [22] and also in FRDA fibroblasts (this manuscript). These data demonstrate the difficulty in obtaining stable cell line deficient in frataxin and could explain the paucity of long term stable models, necessary for pharmacological screening. As the long term persistence of frataxin deficiency was not tested in the stable RNAi model [47], we do not know if this adaptative mechanism is specific to our antisense strategy or is a more general feature characteristic of frataxin deficient cell lines.

## Conclusion

The present work demonstrates the feasibility of a cell-based high-throughput screening assay in Friedreich ataxia but highlights the necessity of developing long-term robust cellular model that reproduce the physiopathology of the disease. Furthermore, the readout for the screen should be as specific as possible, in accordance to technical limitations.

## Competing interests

The authors declare that they have no competing interests.

## Authors' contributions

NCC carried out the characterization of the cellular model, participated in the pharmacological screening and drafted the manuscript. HS carried out the development of the cellular model and participated to the characterization. PV carried out the pharmacological screening. LR participated to the characterization of the cellular model. MH and JH conceived the screening strategy. PR carried out the biochemical analyses. MK and HP conceived the study, and participated in its design and coordination and drafted the manuscript. All authors read and approved the final manuscript.

## Pre-publication history

The pre-publication history for this paper can be accessed here:


